# Light competition drives herbivore and nutrient effects on plant diversity

**DOI:** 10.1038/s41586-022-05383-9

**Published:** 2022-11-02

**Authors:** Anu Eskelinen, W. Stanley Harpole, Maria-Theresa Jessen, Risto Virtanen, Yann Hautier

**Affiliations:** 1 Department of Physiological Diversity, Helmholtz Centre for Environmental Research–UFZ, Leipzig, Germany; 2 German Centre for Integrative Biodiversity Research (iDiv) Halle-Jena-Leipzig, Leipzig, Germany; 3 Department of Ecology and Genetics, University of Oulu, Oulu, Finland; 4 Institute of Biology, Martin Luther University Halle-Wittenberg, Halle, Germany; 5 Department of Community Ecology, Helmholtz Centre for Environmental Research–UFZ, Halle, Germany; 6 Ecology and Biodiversity Group, Department of Biology, Utrecht University, Utrecht, The Netherlands; 7 Present Address: Department of Ecology and Genetics, University of Oulu, Oulu, Finland

**Keywords:** Community ecology, Biodiversity

## Abstract

Enrichment of nutrients and loss of herbivores are assumed to cause a loss of plant diversity in grassland ecosystems because they increase plant cover, which leads to a decrease of light in the understory^1–3^. Empirical tests of the role of competition for light in natural systems are based on indirect evidence, and have been a topic of debate for the last 40 years. Here we show that experimentally restoring light to understory plants in a natural grassland mitigates the loss of plant diversity that is caused by either nutrient enrichment or the absence of mammalian herbivores. The initial effect of light addition on restoring diversity under fertilization was transitory and outweighed by the greater effect of herbivory on light levels, indicating that herbivory is a major factor that controls diversity, partly through light. Our results provide direct experimental evidence, in a natural system, that competition for light is a key mechanism that contributes to the loss of biodiversity after cessation of mammalian herbivory. Our findings also show that the effects of herbivores can outpace the effects of fertilization on competition for light. Management practices that target maintaining grazing by native or domestic herbivores could therefore have applications in protecting biodiversity in grassland ecosystems, because they alleviate competition for light in the understory.

## Abstract (web summary)

Competition for light can cause plant diversity loss in grassland ecosystems when fertilized or herbivores are excluded, and experimentally restoring light can mitigate this biodiversity loss.

## Main

Anthropogenic nutrient enrichment and human-induced shifts in herbivore pressure are two major drivers of global change that markedly alter the composition, diversity and functioning of terrestrial plant communities^3–5^. Increased nutrient supply due to eutrophication has been connected to a strong and persistent loss of diversity^6,7^, and herbivory by grazing mammals is known to be one of the key factors that maintains diversity^2,8,9^, as described already by Charles Darwin in his *On the Origin of Species* in 1859: “No wonder that, as soon as the land was enclosed, it became thickly clothed…”^10^. The assumed central mechanism that drives these responses and links the effects of nutrients and herbivory on diversity is competition for light^1–3,11^. Fertilization disproportionally promotes the growth of taller plants with more canopy cover and better access to light, at the expense of shorter plants and seedlings in the understory, and this leads to reduced diversity^12,13^. The asymmetry in competition for light stems from the fact that light as a resource is unidirectional^13,14^. By contrast, by consuming vegetation and selectively targeting taller species that are superior in the competition for light, herbivores can directly reduce canopy cover and increase the availability of light for shorter plants^1,15^. Herbivory therefore has the potential to alleviate competition for light and maintain diversity^2,3^.

Testing the role of light in maintaining diversity requires direct experimental tests in which light—the limiting resource—is added into the understory of plant communities where competition for light is strongest. However, most studies use different indirect ways to address the role of competition for light, such as unmanipulated light measurements, tiebacks or neighbour removals^3,9,13,16^, which, for methodological reasons, may produce misleading results. For example, neighbour removal could release nutrients from roots and alter temperature and humidity, and might not reflect light as a causal factor. The strongest direct evidence so far comes from a controlled greenhouse experiment﻿, which showed that adding light by lamps to the understory of plant communities prevented the loss of species that otherwise resulted from fertilization^12^. However, these results have not been confirmed in natural field conditions, with more complex communities and with herbivores that are predicted to interact with nutrient effects on competition for light^17,18^.

**Fig. 1:**
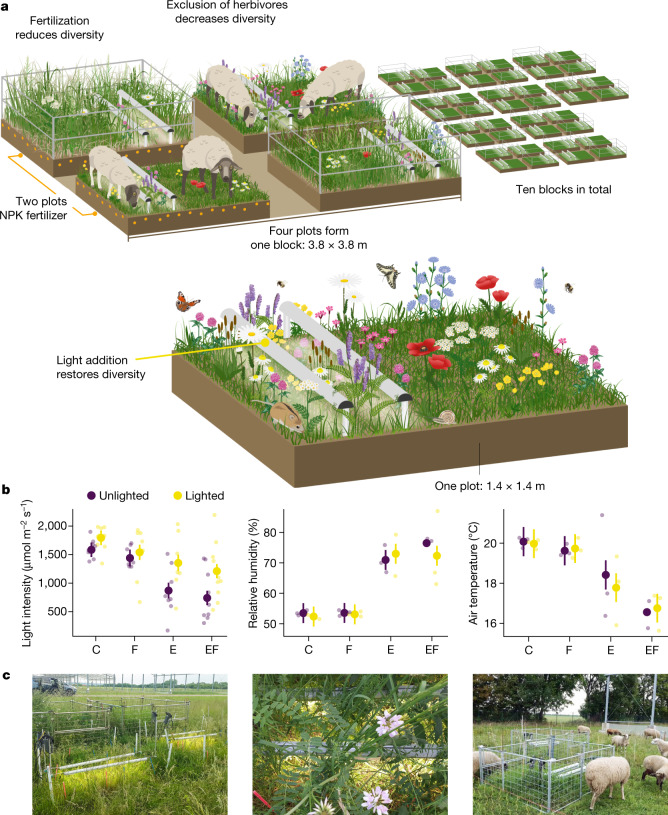
Illustration of the eDiValo experiment. **a**, Experimental design and predictions of the effects of treatments. See Methods for more details on the NPK fertilizer, herbivore exclosures, and lamps used for light addition. **b**, Effects of treatments on light quantity, relative humidity and air temperature. Values are mean ± s.e.m. derived from linear mixed-effects (LME) models, in which parameter significance was assessed by *F*-tests (two-tailed). For sample sizes and statistics, see Extended Data Table 1. Faded dots show individual data points. C, control; F, fertilization; E, exclosure; EF, exclosure and fertilization. **c**, Photos of eDiValo experimental design (left), lamps bringing light to small understory plants (middle) and a flock of sheep grazing in an experimental block (right). Photo credit: A.E. Images in **a** were created by G. Rada (iDiv, Media and Communications).

Here, we experimentally manipulated light, herbivory and nutrient supply, in a full-factorial design, to test the direct causal role of light limitation in driving plant diversity loss from eutrophication and loss of herbivory in a natural, species-rich grassland. We installed modern light-emitting diode (LED) lamps below the plant canopy to provide light to plants in the understory, where light should be needed most. We combined the light-addition treatment with fertilization and exclosures that prevented grazing by sheep to test the responses of plant community richness and diversity (Fig. 1a,c and Methods). Our LED lamps, with a spectrum mimicking natural sunlight, increased the availability of light in the understory compared to ambient levels (a 57% increase, on average, in the quantity of light in fertilized and fenced plots by light addition; Fig. 1b). Furthermore, our light-addition treatment had no detectable effects on humidity and air temperature near the soil surface (Fig. 1b and Extended Data Table 1)﻿, and caused minimal disturbance (Methods). Our experiment, which we term ‘eDiValo’ (ecological effects of light (‘valo’ in Finnish) on diversity) was conducted in grazed pastures at the Global Change Experimental Facility (GCEF) in Bad Lauchstädt, Germany, where species-rich natural grassland vegetation (approximately 23 vascular plant species in a 0.25-m^2^ area) was exposed to short-time high-intensity sheep-grazing events two to three times each growing season. We measured species richness (because richness should be sensitive to rare species becoming extinct) and Shannon diversity (because diversity should be sensitive to species becoming less abundant before they become extinct), total live plant and litter cover and plant functional traits in 0.5 × 0.5-m plots.

**Fig. 2:**
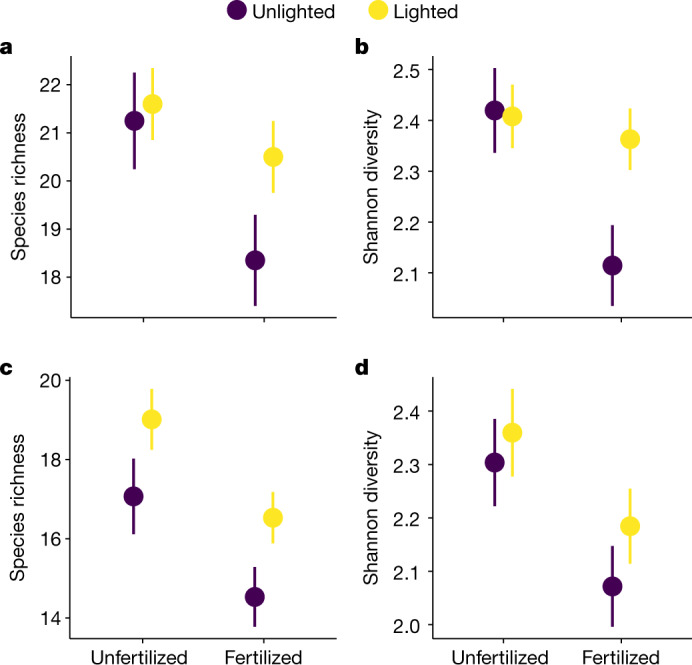
Addition of light mitigates the loss of plant diversity due to nutrient addition in 2017 but not in 2019. **a**–**d**, Effects of fertilization and light addition on species richness (**a**) and Shannon diversity (**b**) in 2017 and on species richness (**c**) and Shannon diversity (**d**) in 2019. Values are mean ± s.e.m. derived from LME models, in which parameter significance was assessed by *F*-tests (two-tailed). In 2017, *n* = 20 for quadrats from which species richness and Shannon diversity were estimated; in 2019: fertilized, unlighted and lighted, *n* = 22; unfertilized, unlighted and lighted, *n* = 18. For statistics, see Methods and Extended Data Tables 2 and 5. In 2017, herbivore exclusion treatment was not yet applied. In 2019, data were pooled across herbivore exclusion treatment to show fertilization effects more clearly; for a full presentation of treatments, see Extended Data Fig. 2. These results are from our eDiValo field experiment; see Fig. 1 and Methods.

First, in 2017, we tested whether adding light could offset the negative effect of fertilization on richness and diversity. During the 2017 growing season, we prevented grazing in the whole experimental area to allow fertilization effects to develop. Fertilization rapidly decreased species richness and species diversity, measured as Shannon diversity, in 2017 (by 9.3% and 7.3%, respectively; Fig. 2a,b and Extended Data Tables 2 and 3). Notably, restoring light to plants in the understory of the fertilization treatment offset the loss of diversity (fertilization × light addition interaction on Shannon diversity; Fig. 2b and Extended Data Table 2). Although light addition reduced the loss of species richness (Fig. 2a and Extended Data Table 2), the interaction between light addition and fertilization was weaker on species richness than on Shannon diversity, suggesting that light addition promoted more equal abundances, whereas richness—through either extinctions or gains—was slower to respond. Our findings confirm the mechanism that was reported in a previous controlled greenhouse experiment, in which added light similarly rapidly reversed the loss of species resulting from nutrient addition^12^. This result is consistent with the hypothesis of competition and diversity in herbaceous vegetation^1^, the theory of resource competition^15^ and quantitative models that address the asymmetric nature of competition for light^13,14^. Our study provides the first—to our knowledge—direct experimental demonstration that competition for light is a central mechanism that leads to the loss of plant diversity in conditions of nutrient enrichment, in species-rich real-world grassland communities that experience varying environmental conditions and complexities of trophic interactions.

**Fig. 3:**
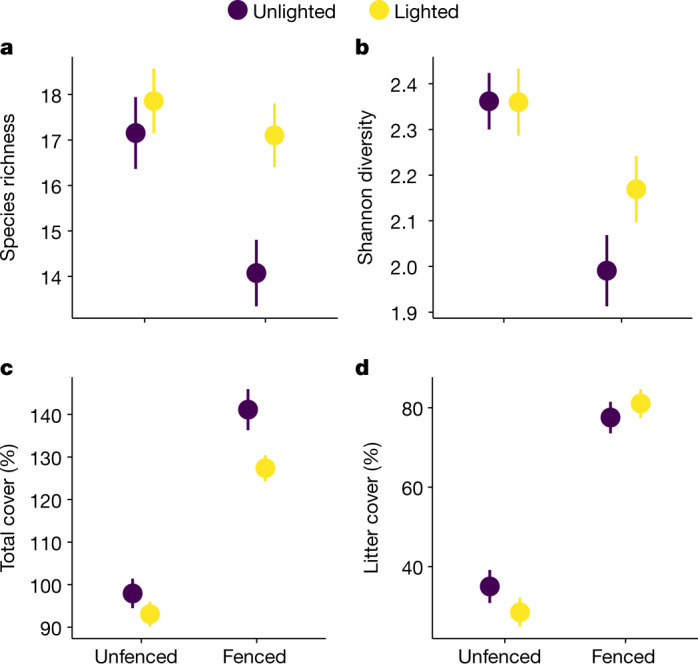
Addition of light mitigates the loss of plant diversity due to herbivore exclusion in 2019. **a**–**d**, The effects of fencing and light addition on species richness (**a**), Shannon diversity (**b**), total cover (**c**) and litter cover (**d**). Both total and litter cover were visually estimated from 0.5 × 0.5-m plots (see Methods). Values are mean ± s.e.m. derived from LME models, in which parameter significance was assessed by *F*-tests (two-tailed); *n* = 20 for quadrats from which species richness, Shannon diversity, total cover and litter cover were estimated. For statistics, see Methods and Extended Data Tables 2 and 5. For a full presentation of the treatments, see Extended Data Figs. 2 and 3. These results are from our eDiValo field experiment; see Fig. 1 and Methods.

Second, after the first experimental year, we extended our experimental design to include herbivory by sheep, and erected fenced herbivore exclosures randomly around half of the plots that were established in 2017 (Fig. 1a). We simultaneously removed the temporary fence around the whole experimental area and allowed the grazing of sheep in unfenced plots. In 2019, we tested whether light addition could offset the negative effect of herbivore exclusion on richness and diversity and whether light, nutrients and herbivory interact. Herbivore exclusion decreased species richness by 12.5% and Shannon diversity by 11.7%, independent of fertilization (Fig. 3a,b and Extended Data Tables 2 and 4) and consistent with previous studies^3,8^. At the same time, herbivore exclusion increased total cover (Fig. 3c) and decreased light (Fig. 1b). These results indicate that herbivory is a dominant factor controlling light availability and plant diversity. Our key finding was that experimental addition of light in the understory mitigated the loss of richness and diversity due to the herbivore exclusion (exclosure × light interactions on richness and Shannon diversity; Fig. 3a,b, Extended Data Tables 2 and 4 and Extended Data Fig. 2b,c). This provides experimental evidence that herbivores maintain diversity by alleviating competition for light. Extirpation of large mammalian herbivores^5,19^ may therefore contribute to diversity loss in plant communities through increased competition for light.

Fertilization had a negative main effect on both richness and diversity in 2019 (leading to decreases of 12.7% and 8.2%, respectively, similar magnitudes to 2017; Extended Data Tables 2 and 4 and Fig. 2c,d); however, light-addition effects in fertilized plots compared to 2017 were transitory and did not restore diversity in 2019 (no significant fertilization × light interaction; Extended Data Tables 2 and 4), in contrast to the effects of light addition in herbivore exclosures (Fig. 3a,b and Extended Data Fig. 2a,b). The negative main effect of fertilization on plant diversity was independent of changes in total cover and light availability, which were not affected by fertilization (Figs. 1b and 3c and Extended Data Fig. 2c). As a result, addition of light in fertilized plots did not restore diversity. By contrast, in the continued absence of herbivory, control of light competition shifted from bottom-up effects of fertilization to top-down control by consumers that regulated total cover, light availability, competition for light and diversity. Our results suggest that herbivore-mediated processes linked to succession can outpace the effects of fertilization on competition for light. However, mechanisms other than competition for light could explain the negative main effect of fertilization on diversity. For example, litter cover was higher in fertilized than in unfertilized plots (64.6% versus 44.9%, respectively), which could have contributed to this negative effect (main effect of fertilization; Extended Data Tables 2 and 4 and Extended Data Fig. 3b). As predicted by theory^15^, supplying multiple nutrients could also have reduced diversity without increasing total cover and light limitation by reducing the dimensionality of belowground nutrient trade-offs^20^. Fertilization could also exert direct toxic effects on seedling richness, reducing diversity through decreased recruitment opportunities^21^. Our results are consistent with these previous findings and suggest that herbivores can outpace the adverse effects of fertilization on diversity when they occur through intensifying competition for light and affecting litter, but not when they occur through filling nutrient niches in the soil or by direct toxic effects on seedlings.

Adding light, however, did not completely offset the negative effect of herbivore exclusion on Shannon diversity. One explanation is that additional light did not fully alleviate light limitation in the understory inside herbivore exclosures (Fig. 1b). Alternatively, besides competition for light, other factors could also have contributed to the loss of diversity inside herbivore exclosures. In our experimental plots, herbivore exclusion more than doubled the amount of undecomposed plant litter (32.8% cover outside exclosures versus 78.7% cover inside exclosures, Fig. 3d), which can decrease diversity through reduced opportunities for plant recruitment from seed^22^. Several other factors can also change as a result of herbivore exclusion and affect diversity^23^. We showed that herbivores exhibited the strongest control on litter and total cover (Fig. 3c,d and Extended Data Fig. 3b), which modified the abiotic environment, leading to a lower air temperature (2.5 °C and 12.2% lower on average) and higher levels of humidity (35% higher) inside exclosures than in grazed plots (Fig. 1b). In addition, increasing the levels of light slightly reduced the total cover inside unfertilized exclosures (Fig. 3c and Extended Data Fig. 2c). This result is counterintuitive, but could be due to changes in species composition, more-even distribution of species in the canopy and/or reduced cover by dominant species in this treatment combination. Overall, our results show that changes in understory plant life in ungrazed conditions include a suite of factors, all of which may be important for plant performance^23,24^.

**Fig. 4:**
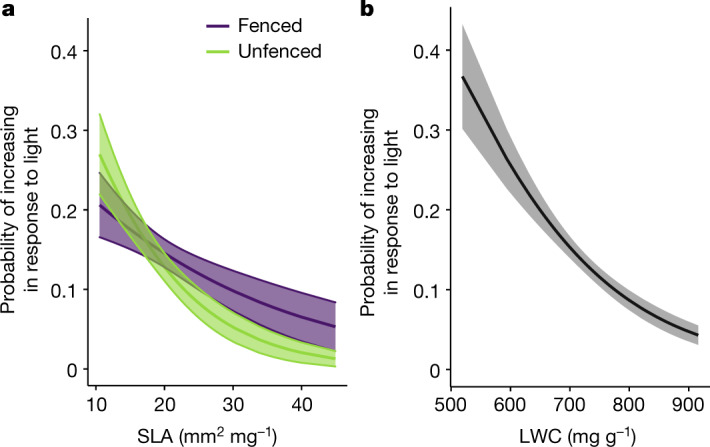
Plants with a lower specific leaf area and lower moisture content are more likely to increase with the addition of light. **a**,**b**, Probability that individual species increase in response to light addition as a function of specific leaf area (SLA; **a**) and leaf water content (LWC; **b**) in 2019. Fitted lines are from generalized linear mixed-effects (GLME) models with a binomial error structure, in which parameter significance was assessed by *χ*^2^-tests (two-tailed; see Methods): **a**, exclosure *χ*^2^ = 0.45, *P* = 0.50; SLA *χ*^2^ = 52.24, *P* < 0.0001; exclosure × SLA *χ*^2^ = 8.08, *P* = 0.0045, **b**, LWC *χ*^2^ = 87.18, *P* < 0.0001. Shaded areas represent 95% confidence intervals; *n* = 1,580 species observations for fenced and unfenced SLA and *n* = 3,240 species observations for LWC. In the models, species were nested within plots (40) that were nested within blocks (10). Fenced, no exclosure; Unfenced, exclosure. Species with a lower SLA benefit more from light addition in unfenced plots compared to fenced plots (exclosure × SLA interaction). These results are from our eDiValo field experiment; see Fig. 1 and Methods.

Finally, we assessed which plant functional traits made species more or less responsive to light addition under fertilization and herbivore exclusion. Short species (size-related trait) and species with low light interception (trait related to conservative resource-use; a small specific leaf area, SLA) should be at a competitive disadvantage under low light conditions^25–27^, and should therefore benefit the most from light addition. Consistent with this prediction, species with a lower SLA and lower leaf water content (LWC) had a higher probability to increase from unlighted to lighted subplots; that is, were more likely to benefit from light addition (Fig. 4 and Extended Data Fig. 4). By contrast, short species did not benefit from light addition (see Methods). It is possible that SLA and LWC better integrate plant responses to light during the whole life cycle, including seedling and juvenile stages, even if height is more important for the competitive ability of adult plants^24,28^.

Anthropogenic nutrient enrichment and changes in the grazing practices of livestock and native herbivores are among the most influential global-change factors that can decrease or rescue the biodiversity of global grasslands^3,6,9,29^. Understanding the ecological mechanisms by which nutrients and consumers operate is fundamental for our ability to maintain and manage biodiversity, and to develop better conservation actions in the Anthropocene. By supplying light to understory plants, our experimental results here provide direct evidence of the role of competition for light as a mechanism that decreases diversity under fertilization and the absence of mammalian herbivory in natural grasslands. Our findings especially highlight the importance of grazing by native and domestic herbivores as factors that foster higher biodiversity. Carefully planned livestock management practices, protection of native herbivores and re-establishing large mammalian herbivores to areas from where they have been extirpated (that is, rewilding) may therefore be key conservation strategies in the Anthropocene^30,31^, because they could promote plant diversity by alleviating competition for light.

## Methods

### Study site and future climate treatment

Our study site is located at the Bad Lauchstädt Field Research Station, Bad Lauchstädt, Germany (51° 22060 N, 11° 50060 E), which belongs to the Helmholtz Centre for Environmental Research–UFZ. Long-term mean annual precipitation in the area is 489 mm and the mean annual temperature is 8.9 °C (ref. ^32^). During 2018 and 2019, Europe experienced a record-setting drought that was especially severe in 2018 (refs. ^33,34^); the mean annual precipitation at our study site in 2018 and 2019 was 254 mm and 353 mm, respectively, whereas 2017 was a more normal year, with a mean annual precipitation of 403 mm. Mean annual temperatures were above average: 2017, 10.5 °C; 2018, 10.8 °C; 2019, 11.2 °C (data from the weather station at the Bad Lauchstädt field station). The soils in the study area are fertile Haplic Chernozem type^32,35^.

Our eDiValo experiment was conducted in the GCEF, which was designed to investigate climate change effects under different land-use scenarios^32^. We used 10 ‘extensively’ used pastures of the GCEF in our experiment; that is, 384-m^2^ (16 × 24 m) areas of grassland (hereafter called ‘pastures’) that were grazed by a flock of 20 sheep 2–3 times each year. Grazing was implemented as short-time high-intensity grazing events, each lasting 24 h (ref. ^32^). This type of high-intensity but short-term grazing is considered better in maintaining species richness as it gives plants more time to recover between grazing events^36^. It is also a recommended management type for nature conservation areas in Germany^37^. Vegetation in the pastures was species-rich grassland vegetation that is typical of drier regions of central Germany^32,38^. The whole GCEF was fenced to exclude native large mammalian herbivores (for example, deer); however, European hare (*Lepus europaeus*), wood mice (*Apodemus sylvaticus*) and voles (*Microtus arvalis*) are common at the site.

Our experimental design was originally intended to test the dependence of light competition on nutrient and herbivory under current and future climatic scenarios. Although we included both climate treatments in our data, climate was never significant for richness and Shannon diversity, either alone or in interaction with other factors, and our focus was therefore on the other treatments. Five of the above random pastures received future climatic treatment which was based on different dynamic regional climate models for Germany, all predicting an increased mean temperature by approximately 2 °C year-round, strongly decreased summer precipitation and slightly increased spring and autumn precipitation (https://www.regionaler-klimaatlas.de/) (ref. ^32^). Passive night-time (after sunset and before sunrise) warming through the use of roller blinds attached to the GCEF roof and eastern and western wall structures was used to increase the air temperature. In each spring (1 March–31 May) and autumn (1 September–30 November), future climate plots received 110% of the ambient rainfall and in the summer (1 June–31 August), they received 80% of the ambient rainfall. The precipitation treatment was adjusted weekly and compensated for a possible night-time reduction in rainfall due to temperature treatment. A detailed description of the future climate treatment is provided in a previous report^32^.

### Fertilization, herbivore exclusion and light addition

We first tested whether adding light can offset the negative effect of fertilization on plant diversity. In May 2017, we established a full-factorial experiment of fertilization and light addition. Within each 10 pastures (5 in ambient climatic conditions, 5 in future climatic conditions), we established 4 plots of 1.4 × 1.4 m, separated by a 1-m buffer zone (hereafter called ‘blocks’), in total 40 plots and 10 blocks. At the time the experiment was established, vegetation in the whole experimental area (that is, in a block of 4 plots and the surrounding 1-m area) was trimmed to a height of 5 cm to make conditions uniform and the whole area was temporarily fenced to let the experiment establish and fertilization effects develop. The temporary fence was removed in August when the herbivore exclusion treatment was started. Therefore, there was no grazing by sheep in the experimental plots in the summer of 2017. Two randomly chosen plots received fertilizer treatment and two were controls. For the former (fertilizer-treatment plots), slow-release granular NPK fertilizer (a mixture of Haifa Multicote 2 M 40-0-0 40% N; Triple Super Phosphate (TSP) 45% P_2_0_5_; and potassium sulfate fertilizer 50% K_2_O, 45% SO_3_) was added twice per growing season, in a total of 10 g N, 10 g P and 10 g K per m² (see ref. ^3^ for a similar protocol that is used in grasslands worldwide). In 2017, the first fertilization was done at the beginning of June right after establishing the experiment and the second fertilization was done at the beginning of July. In the subsequent years, the first fertilization was done at the beginning of the growing season (late March–April) and the second fertilization was done in June. In 2019, two previously unfertilized plots were accidentally fertilized and were thereafter treated as fertilized plots. ﻿

To manipulate light, 1.4 × 1.4-m plots were further divided into two subplots, 0.7 m × 1.4 m each, and one of these was randomly assigned to the light-addition treatment, resulting in 80 subplots (Fig. 1). We installed two 120-cm-long and 3.5-cm-wide recently developed LED lamps (C65, Valoya) parallel to each other and at a 28-cm distance from each other to each light-addition subplot. To increase light for the small understory plants that are the most likely to suffer from competition for light, we installed the lamps 10 cm above the smallest plants. The lamps were gradually uplifted over the course of the growing season to follow the growth of the smallest plants. As our light-addition treatment was intended to mimic natural sunlight (that is, making a gap in a dense vegetation and allowing the sunshine in), we chose the spectrum of the lamps to include all wavelengths of sunlight, including small amounts of ultraviolet and infrared. Each lamp added roughly 350–400 µmol and did not alter the air or aboveground soil surface temperature (Fig. 1b), which is an improvement on previous studies^12^. Each year, we added light during the active growing season: the lamps were switched on early in the spring (March–April), when temperatures were clearly above zero, and switched off and removed when temperatures dropped close to zero in November–December and aboveground plant parts had died and formed litter. Each day, the lamps were set to switch on two hours after sunrise, and to switch off two hours before sunset, and when the temperature exceeded 28 °C to prevent overheating. We did not install unpowered lamps to unlighted plots because our modern, narrow LED lamps caused minimal disturbance (see below) and no heating (Fig. 1b), and because unpowered lamps would have added an artefact in that they create shade that does not occur when the lamps are on in lighted plots.

At the end of August 2017, after running the fertilization–light-addition experiment for one growing season, we expanded the experiment by implementing the herbivore exclusion treatment in a full-factorial combination with the other treatments. Two of the previously established 1.4 m × 1.4-m plots, one with and one without the fertilization treatment, were randomly allotted to the herbivore (sheep) exclusion treatment and fenced with rectangular metal fences of 1.8 m × 1.8 m, 82 cm height and 10 cm mesh size. At the same time, the temporary fence established in May 2017 was removed from around the whole experimental area, allowing the grazing of sheep in unfenced plots. The fences did not exclude mice, voles and hares. For the time of each grazing event, lamps in grazed subplots were removed and switched off in the ungrazed subplots. Uplifting the lamps from grazed plots did not cause disturbance because vegetation in grazed plots was always short and did not reach above the lamps. Inside exclosures, lamps were always kept in place during the growing season, and plants could freely grow around and above them.

### Plant community and trait sampling

In July 2017, we established 50 cm × 50-cm permanent quadrats in every subplot for plant community sampling. We visually estimated the per cent areal cover for all species occurring in the quadrats, and litter cover, from the beginning of June to mid-June 2019, when the vegetation was at its peak biomass. The 2017 sampling happened later, in mid-July, because vegetation in all plots and surrounding areas was trimmed to a height of 5 cm at the time of the establishment of the experiment at the end of May, and it took later for vegetation to reach its peak biomass. In 2018, the effects of drought were devastating, and most plants had senesced or died before the planned sampling date; we therefore omitted the year 2018. At the beginning of each growing season—that is, when the lamps were installed and switched on—there was very little live biomass in the plots, and the maximum height of existing plants was approximately 5 cm (in all plots). During the peak biomass the maximum plant height was up to approximately 1 m; however, it varied greatly between the treatments and was especially low in grazed plots. All vegetation surveys were done by the same trained and experienced person with a minimum estimate threshold of 0.1%. We used plant cover data to calculate species richness and Shannon diversity.

In May–June 2020, we measured plant height (centimetres), SLA (leaf area in square millimetres per milligram of dry mass), foliar C:N (based on the per cent C and N in plant leaves) and LWC (leaf water content as 1,000 − LDMC (the ratio of leaf dry mass to saturated fresh mass), expressed as milligrams per gram^39^) for most species occurring in the experimental plots, and complemented the trait data from the TRY Plant Trait Database^40–42^ (v.5.0; https://www.try-db.org/TryWeb/Home.php) and for one species one trait value from another source^9^. The trait data were collected from seven to ten individuals per species from the study site or close areas; the collection and handling followed standard protocols^39^. We chose these traits because they are widely documented to be associated with responsiveness to soil nutrients, herbivory and light^9,26,27,43–46^. We used all traits as, although they partially reflect similar ecological adaptations (for example, leaf economics spectrum^43^), they could also potentially reflect independent and distinctive processes, and differently mediate the responses of species to our treatments. For example, SLA and LWC in our dataset correlated weakly (*r*^2^ = 0.16), but were to a greater extent uncorrelated (Extended Data Table 6), and could function differently, for example, in light capture and drought tolerance^26,39^. In 2017, our trait data covered on average 97.7–98.6% of the total cover in the plots, the value slightly differing depending on the trait as we did not have all traits for all species. Our own trait collections covered on average 96.6–97.6% and TRY data covered on average 0.9–2% of the total cover. In 2019, the whole trait data covered on average 99.5% of the total cover in the plots, again slightly depending on the trait. Our own trait collections covered on average 94.2–96.5% and TRY data covered on average 2.7–5.3% of the total cover.

### Abiotic environmental measurements

We measured several soil and other environmental properties from the experimental plots. Light availability (photosynthetically active radiation; PAR) in unlighted and lighted (under lamps) subplots was measured using LI-190R and LI-250A meters (LI-COR), approximately 7–10 cm under the lamps and 15–20 cm above ground level. We measured light availability from the same distance to the ground in unlighted plots. Measurements of light availability were done in mid-July 2020 on three consecutive cloudless days around noon. Note that in grazed plots, light levels between lighted and unlighted plots are more similar than inside exclosures (Fig. 1), because herbivores keep the vegetation short, and natural sunlight can therefore reach under the lamps where the light measurements were taken. Air temperature and humidity were recorded from unlighted and lighted (under lamps) subplots using loggers (HOBO MX2301A, Onset Computer Cooperation) that were installed approximately 7 cm under the lamps and to the same height from the ground in unlighted plots, and were replicated under different combinations of fertilization, herbivore exclusion and light addition in ambient climatic conditions three times (*n* = 3). The logger data were collected in May 2019 before the effects of drought were visible.

### Statistical analysis

**Extended Data Fig. 1:**
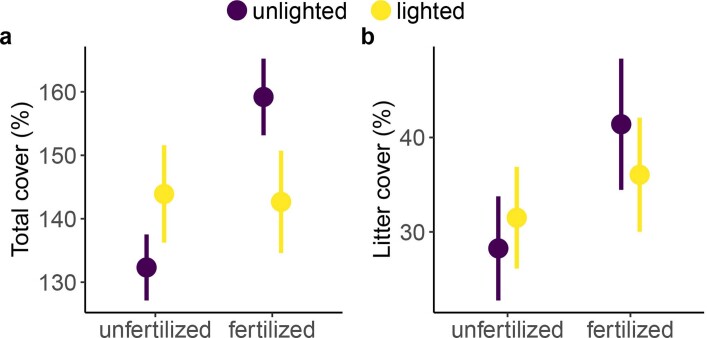
Effects of experimental treatments on total and litter cover in 2017. Effects of fertilization and light addition on total vegetation cover % (**a**) and litter cover % (**b**) in 2017. In 2017, fencing (herbivore exclusion) treatment was not yet applied. Values are means ± s.e.m. derived from LME models where parameter significance was assessed by *F*-tests (2-tailed), *n* = 20 for quadrats from which species richness and Shannon diversity were recorded; for statistics see Methods and Extended Data Tables 2 and 5. In 2017, herbivore exclusion treatment was not yet applied. These results are from ‘eDiValo’ field experiment; see Fig. 1 and Methods.

**Extended Data Fig. 2:**
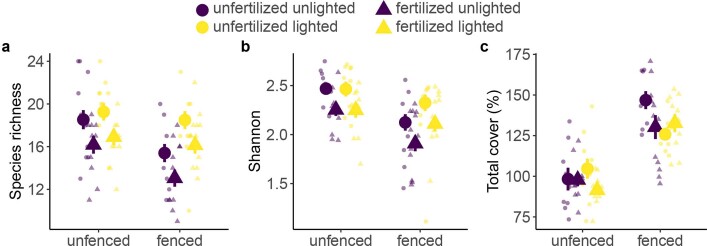
Effects of fencing, fertilization and light addition on species richness, Shannon diversity and total cover in 2019. (Species richness (**a**), Shannon diversity (**b**), and total vegetation cover (%) (**c**). Values are means ± s.e.m. derived from LME models where parameter significance was assessed by *F*-tests (2-tailed); for statistics see Methods and Extended Data Tables 2 and 5. unfenced, unlighted and lighted *n* = 10; fenced, unlighted and lighted *n* = 8; fenced and fertilized, lighted and unlighted *n* = 12; fertilized, unlighted and lighted *n* = 10. Individual data points are shown with faded colour. Sampling unit is a quadrat from which litter was estimated. The different sample sizes result from two previously unfertilized plots being accidentally fertilized in 2019 after which these plots were considered fertilized plots. These results are from ‘eDiValo’ field experiment; see Fig. 1 and Methods.

**Extended Data Fig. 3:**
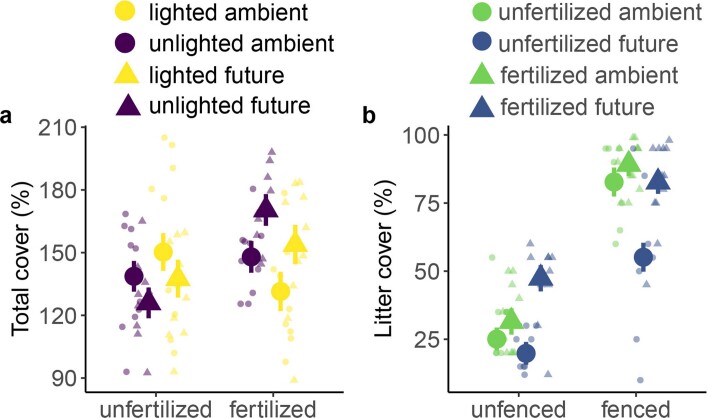
Effects of treatments on total cover in 2017 and on litter cover in 2019. The effects of fertilization, light, and climate on total cover (%) in 2017 (﻿**a**). *n* = 10 for quadrats from which total cover was estimated. The effects of fencing, fertilization, and climate treatment on litter cover (%) in 2019 (**b**). unfenced, ambient and future climate *n* = 10; fenced, ambient and future climate *n* = 8; fenced and fertilized, ambient and future climate *n* = 12; fertilized, ambient and future climate *n* = 10. Sampling unit is a quadrat from which litter was estimated. The slightly unequal samples sizes in 2019 result from two previously unfertilized plots being accidentally fertilized in 2019 after which these plots were considered fertilized plots. In both 2017 and 2019, values are means ± s.e.m. derived from LME models where parameter significance was assessed by *F*-tests (2-tailed); for statistics see Methods and Extended Data Tables 2 and 5. These results are from ‘eDiValo’ field experiment; see Fig. 1 and Methods.

We analysed our data in two steps. First, to test whether competition for light mediates the effect of fertilization on diversity, we analysed the effects of fertilization and light and their interaction on species richness and Shannon diversity using data from 2017, when the herbivore exclusion treatment had not yet been implemented. We also analysed the effects of treatment on total vegetation cover and litter cover. We fit LME models in which diversity (species richness and Shannon diversity), total cover and litter cover, each in their own model, were explained by fertilization, light addition and their interaction (fixed variables). All treatments were categorical variables with two levels (treated and untreated). In each model, subplot was nested within plot, which was nested within block (nested random variable). We simplified the models using the anova() function for model comparison in the nlme and lme4 packages in R (ref. ^47^) (on the basis of log likelihood ratio tests; *P* ≥ 0.05; Extended Data Table 2). This was done to uncover the significance of the main effects and interaction terms, to avoid overparametrization^47,48^ and to provide model-derived parameter estimates for the figures (Extended Data Table 5). However, we also provide full model results that are qualitatively similar to the results of simplified models (Extended Data Tables 3 and 4); therefore, model choice did not affect our conclusions. Climate treatment was included in all original models but was never significant for richness and diversity, and was not considered further. Total cover and litter results for 2017 are reported in Extended Data Figs. 1a,b and 3a). As there was heterogeneity in the variance structure between treatments, we used the varIdent() function in the nlme package in R to allow each treatment combination to have a different variance. Model fit was inspected using model diagnostic plots in the package nlme. In the full design with climate included, the number of replicates per treatment combination was ten.

Second, to include herbivore exclusion to the experimental design and to test whether competition for light mediates the effect of herbivore exclusion on diversity, and whether competition for light, herbivory and fertilization interact, we analysed the effects of herbivore exclusion, fertilization, light and their interactions on species richness and Shannon diversity using data from 2019. All treatments were categorical variables with two levels (treated and untreated). We also analysed the effects of treatment on total vegetation cover and litter cover. We fit similar models to those described above, except that herbivore exclusion was an additional fixed factor in the models. We simplified the models, used the varIdent() function to account for heteroscedasticity and checked the model fit using model diagnostic plots, as above. Climate treatment was included in all original models but was significant for litter cover only, and was not considered further. In the full design with climate included, the number of replicates per treatment combination was five.

**Extended Data Fig. 4:**
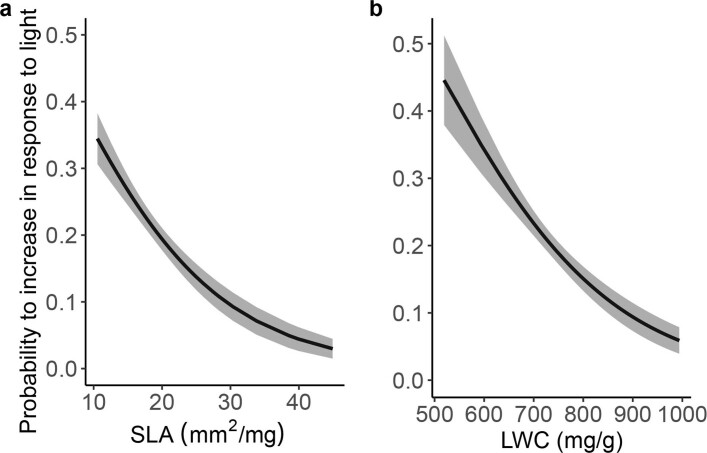
Effects of traits on responses to light in 2017. Probability of individual species to increase in response to light addition as a function of SLA (**a**), and LWC (**b**) in 2017. Fitted lines are from GLME models with a binomial error structure where parameter significance was assessed by χ^2^-tests (2-tailed, see Methods): (a) SLA χ^2^ = 76.21, *P* < 0.0001, (b) LWC χ^2^ = 72.02, *P* < 0.0001. Shaded areas represent 95 % confidence intervals, *n* = 2440 species observations for SLA and LWC. SLA, specific leaf area; LWC, leaf water content. These results are from ‘eDiValo’ field experiment; see Fig. 1 and Methods.

To further assess which plant traits increased the probability of species benefiting from the addition of light, we first created a binary response variable: those species that increased from unlighted to lighted plots (that is, had a higher value in a lighted than an unlighted plot) were given a value of 1 and those that did not were given a value of 0. This response variable takes into account rare species that emerged or persisted in the lighted plots but were absent in the unlighted plots (that is, species gains and losses) and changes in small, subordinate species (those that are likely to benefit from light addition) with small but consistently trait-dependent changes in response to light. It is also in line with our species richness analyses, as species gains and losses ultimately determine richness responses. We did not use different indexes (for example, lnRR or RII) because these could not handle multiple zero values and species losses or gains (that is, species having zero cover in either unlighted or lighted subplots). Second, we fit GLME models with a binomial error structure (family = “binomial”, link = “logit”) in which a probability of a species increasing from unlighted to lighted plots was explained by categorical experimental treatments (fertilization, herbivore exclusion and their interactions), traits (SLA, height, LWC, foliar C:N), and interactions between the treatments and traits. Each trait was analysed in its own model as some of the traits were correlated (Extended Data Table 6), and to avoid overly complex models and overparametrization^47,48^. We included all species for which we had traits in the models. As we calculated the increase in cover from unlighted to lighted plots, our smallest experimental unit in trait analyses was a plot (not a subplot, unlike in other analyses). As there were several species in the same plots, we nested species within plots, and plots within blocks. We similarly simplified the models to include only significant variables (on the basis of *χ*^2^ tests; *P* ≥ 0.05). We did not include a crossed random effect for species in the models because the full models with a more complex random structure did not converge; however, when we refitted the simplified models with a crossed random effect for species, we found that the models converged (with scaled data) and that the significance of the effects remained qualitatively the same. Climate was included in all original models but was never significant. In addition, C:N and height did not predict the responsiveness of species to light in either year (*P* ≥ 0.13 for both); results are therefore not shown. In the full design with climate included, the number of replicates per treatment combination was five; however, the number of observations was greater (see Fig. 4 and Extended Data Fig. 4). To make sure that our results for SLA and LWC were not influenced by whether they were analysed in separate models or in the same model, or by the order in which they were in the models, we also performed analyses in which both SLA and LWC were included (in both orders). Results remained qualitatively similar and are not discussed further.

Furthermore, to check whether our trait results were driven primarily by species gains and losses or changes in abundance, we ran additional trait analyses for which we calculated the change in cover between lighted and unlighted subplots (cover in lighted subplot − cover in unlighted subplot), and analysed the ‘change’ with otherwise similar trait models to those described above, except that we used Gaussian error structure. With this index, which gives a disproportionate importance to the abundant species, we found that traits were poor predictors of changes in cover between lighted and unlighted plots (all interactions were non-significant, *P* > 0.05, except for a marginally significant C:N × fertilization interaction in 2017 that was no longer visible in 2019; results not shown; codes and data available in the Dryad repository). We also analysed presence–absence-based species losses and gains. In these models, each species was given a value of 1 when it was present in the lighted subplot but absent from the unlighted subplot; otherwise, these models were similar to the binomial trait models described above. These models produced, to a large extent, similar results to our models using the probability of increase in response to light as a response variable (results not shown; codes and data available in the Dryad repository). These additional analyses and results support using the probability of increase in response to light as our response variable, rather than abundance-based metrics, as it includes both gains and losses and abundance aspects, and is therefore a general test that is well suited to assessing species gains and extinctions and changes in subordinate species.

All statistical analyses were performed using R v. 4.0.0 (ref. ^49^). We used the nlme package (v.3.1.147) for LME models^50^, the lme4 package (v.1.1.23) for GLME models^51^, and the car package^52^ for *P* values (v.3.07).

### Reporting summary

Further information on research design is available in the Nature Research Reporting Summary linked to this article.

## Online content

Any methods, additional references, Nature Research reporting summaries, source data, extended data, supplementary information, acknowledgements, peer review information; details of author contributions and competing interests; and statements of data and code availability are available at 10.1038/s41586-022-05383-9.

### Supplementary information

Reporting Summary

## Figures and Tables

**Extended Data Table 1:**
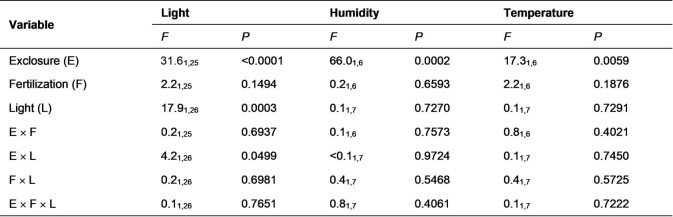
Statistical model results for light quantity, humidity and temperature

Results of LME models testing the effects of exclosure, fertilization, light addition and their interactions on light quantity, humidity and temperature in the experimental plots. In all three models, light-addition treatment (subplots) was nested within plots (that were either fertilized or fenced) that were nested within blocks (that received either ambient or future climate). Sample sizes for light: unfenced, unlighted and lighted *n* = 9; fenced, unlighted *n* = 8, fenced, lighted *n* = 7; fenced and fertilized, lighted and unlighted *n* = 10; fertilized, unlighted *n* = 7; fertilized, lighted *n* = 8. Sampling unit is a quadrat from which light, humidity and temperature measurements were taken. The different sample sizes result from two previously unfertilized plots being accidentally fertilized in 2019, after which these plots were considered fertilized plots. We also missed light measurements from a few plots. Humidity and temperature data were averaged across hour and day; *n* = 3 in all treatment combinations except for fenced and fertilized unlighted, in which *n* = 2.

**Extended Data Table 2:**
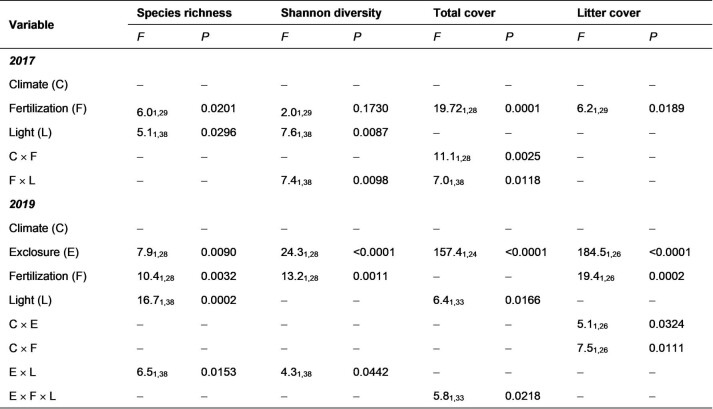
Simplified model results for species richness, Shannon diversity, total cover and litter cover in 2017 and 2019

Simplified LME models testing the effects of climate, fertilization, light addition and their interactions on species richness, Shannon diversity, total cover % and litter cover % in 2017 and the effects of climate, herbivore exclusion (exclosure), fertilization, light addition and their interactions on species richness, Shannon diversity, total cover % and litter cover % in 2019. In all eight models, the light-addition treatment (subplots) was nested within plots (that were either fertilized or fenced) that were nested within blocks (that received either ambient or future climate). We simplified the models to include only significant variables on the basis of log likelihood ratio tests (*P* ≤ 0.05). For full models, see Extended Data Tables 3 (2017) and 4 (2019).

**Extended Data Table 3:**
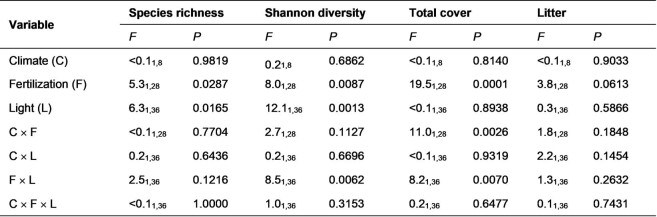
Full model results for species richness, Shannon diversity, total cover and litter cover in 2017

Full LME models testing the effects of climate, fertilization, light addition and their interactions on species richness, Shannon diversity, total cover % and litter cover % in 2017. In all four models, light-addition treatment (subplots) was nested within plots (either fertilized or unfertilized), which were nested within blocks (that received either ambient or future climate). For simplified models, see Extended Data Table 2.

**Extended Data Table 4:**
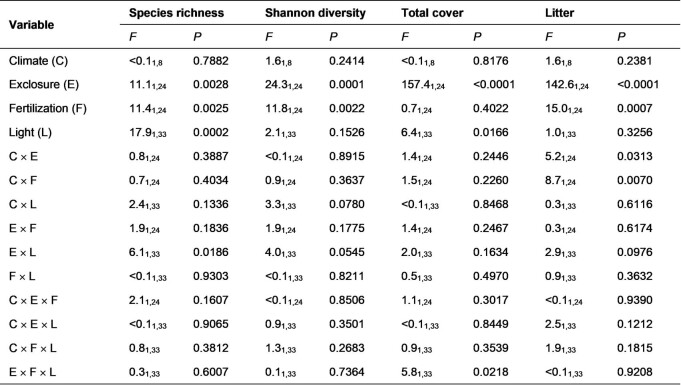
Full model results for species richness, Shannon diversity, total cover and litter cover in 2019

Full LME models testing the effects of climate, herbivore exclusion (exclosure), fertilization, light addition and their interactions on species richness, Shannon diversity, total cover % and litter cover % in 2019. In all four models, the light-addition treatment (subplots) was nested within plots (either fertilized or fenced), which were nested within blocks (that received either ambient or future climate). For simplified models, see Extended Data Table 2.

**Extended Data Table 5:**
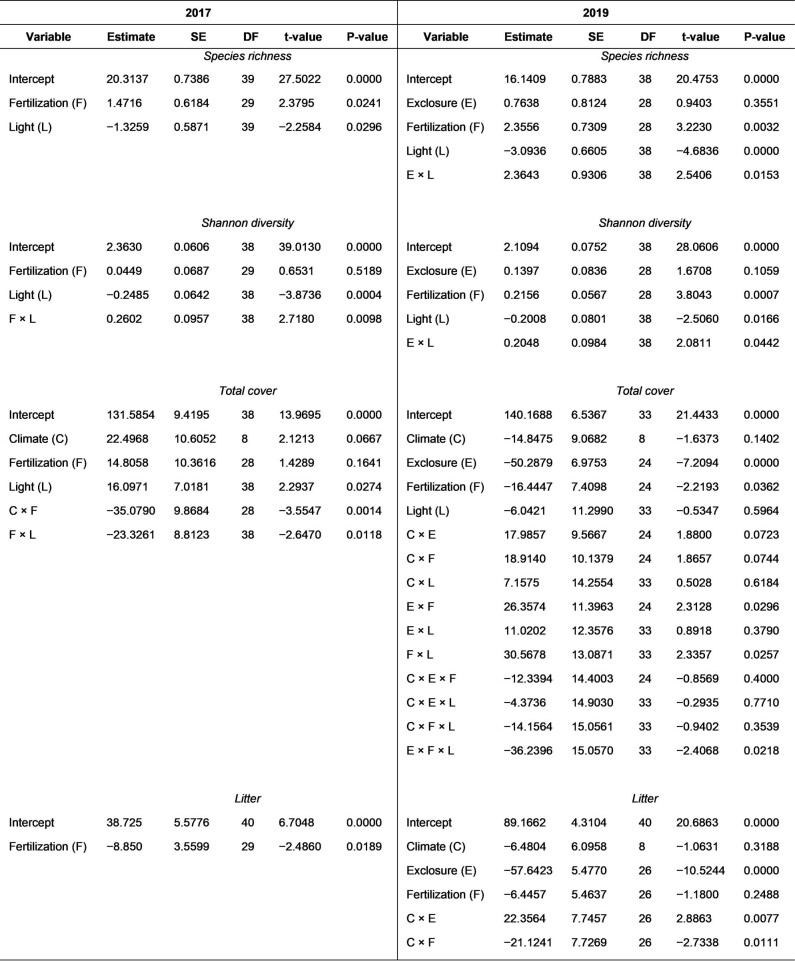
Parameter estimates for species richness, Shannon diversity, total cover and litter cover in 2017 and 2019

Parameter estimates of simplified LME models testing the effects of climate, fertilization, light addition and their interactions on species richness, Shannon diversity, total cover % and litter cover % in 2017 and the effects of climate, herbivore exclusion (exclosure), fertilization, light addition and their interactions on species richness, Shannon diversity, total cover % and litter cover % in 2019. In all four models, the light-addition treatment (subplots) was nested within plots (that were either fertilized or fenced), which were nested within blocks (that received either ambient or future climate). We simplified the models to include only significant variables on the basis of log likelihood ratio tests (*P* ≤ 0.05).

**Extended Data Table 6:**
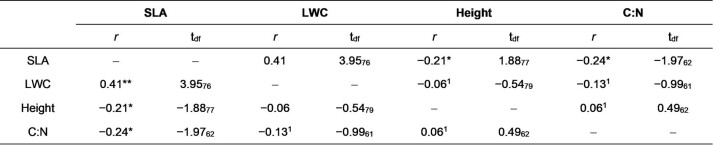
Correlations among individual species’ traits

Pearson correlations (*r*) among specific leaf area (SLA), leaf water content (LWC), height and foliar C:N ratio. The significance of the correlations (six separate models) was assessed by two-tailed t-tests (t). Superscripts denote: 1, *P* > 0.1; *, 0.05 < *P* < 0.1; **, *P* < 0.05.

## Data Availability

The datasets generated and analysed during this study are available in the Dryad repository: 10.5061/dryad.rjdfn2zdm.

## Code availability

The R code and scripts used in this study are available in the Dryad repository: 10.5061/dryad.rjdfn2zdm.
