# Supplementary material for: Light competition drives herbivore and nutrient effects on plant diversity
Source: Nature. 2022 Nov 2;611(7935):301–5. doi: 10.1038/s41586-022-05383-9 (PMC9646529; doi:10.1038/s41586-022-05383-9)
Supplement: Supplementary file 1 — Reporting Summary [file 41586_2022_5383_MOESM1_ESM.pdf]

nature portfolio

Corresponding author(s): Anu Eskelinen

Last updated by author(s): Jun 10, 2022

Reporting Summary

Nature Portfolio wishes to improve the reproducibility of the work that we publish. This form provides structure for consistency and transparency in reporting. For further information on Nature Portfolio policies, see our Editorial Policies and the Editorial Policy Checklist.

Statistics

For all statistical analyses, confirm that the following items are present in the figure legend, table legend, main text, or Methods section.

n/a	Confirmed	
☐	☒	The exact sample size (*n*) for each experimental group/condition, given as a discrete number and unit of measurement
☐	☒	A statement on whether measurements were taken from distinct samples or whether the same sample was measured repeatedly
☐	☒	The statistical test(s) used AND whether they are one- or two-sided<br>*Only common tests should be described solely by name; describe more complex techniques in the Methods section.*
☐	☒	A description of all covariates tested
☐	☒	A description of any assumptions or corrections, such as tests of normality and adjustment for multiple comparisons
☐	☒	A full description of the statistical parameters including central tendency (e.g. means) or other basic estimates (e.g. regression coefficient) AND variation (e.g. standard deviation) or associated estimates of uncertainty (e.g. confidence intervals)
☐	☒	For null hypothesis testing, the test statistic (e.g. *F*, *t*, *r*) with confidence intervals, effect sizes, degrees of freedom and *P* value noted<br>*Give P values as exact values whenever suitable.*
☒	☐	For Bayesian analysis, information on the choice of priors and Markov chain Monte Carlo settings
☐	☒	For hierarchical and complex designs, identification of the appropriate level for tests and full reporting of outcomes
☐	☒	Estimates of effect sizes (e.g. Cohen's *d*, Pearson's *r*), indicating how they were calculated

Our web collection on statistics for biologists contains articles on many of the points above.

Software and code

Policy information about availability of computer code

Data collection	No software was used in the data collection.
Data analysis	All statistical analyses were carried out using R Statistical program version 4.0.0 (https://www.R-project.org/). We used the 'nlme' package for linear mixed effect models (version 3.1.147), the 'lme4' package for generalized linear mixed effects models (version 1.1.23), and the 'car' package for P-values (version 3.07). The R scripts used in the data analyses are available via Dryad repository (https://doi.org/10.5061/dryad.rjdfn2zdm).

For manuscripts utilizing custom algorithms or software that are central to the research but not yet described in published literature, software must be made available to editors and reviewers. We strongly encourage code deposition in a community repository (e.g. GitHub). See the Nature Portfolio guidelines for submitting code & software for further information.

Data

Policy information about availability of data

All manuscripts must include a data availability statement. This statement should provide the following information, where applicable:

- Accession codes, unique identifiers, or web links for publicly available datasets
- A description of any restrictions on data availability
- For clinical datasets or third party data, please ensure that the statement adheres to our policy

The datasets generated and analyzed during the current study are available in Dryad repository (https://doi.org/10.5061/dryad.rjdfn2zdm). Although we collected most trait data ourselves, some trait data was obtained from TRY Trait Plant Database (version 5.0, https://www.try-db.org/TryWeb/Home.php) and originate from Kleyer et al. 2008 and Schroeder-Georgi et al. 2016. One SLA value was obtained from Kaarlejärvi et al. 2017 (https://www.nature.com/articles/s41467-017-00554-z#additional-information).

Field-specific reporting

Please select the one below that is the best fit for your research. If you are not sure, read the appropriate sections before making your selection.

☐ Life sciences ☐ Behavioural & social sciences ☒ Ecological, evolutionary & environmental sciences

For a reference copy of the document with all sections, see nature.com/documents/nr-reporting-summary-flat.pdf

Ecological, evolutionary & environmental sciences study design

All studies must disclose on these points even when the disclosure is negative.

Study description	Our experiment is a full-factorial experiment of climate manipulation, fertilization, herbivore exclusion and light addition, implemented in a nested design. We used 10 grazed pastures of size 400 m2 in our experiment. Five of these pastures received future climatic treatment and five ambient climate treatment. Within each pasture, we applied a full-factorial manipulation of fertilization and herbivore exclusion to four plots of size 1.4 m x 1.4 m . Each plot was further divided into two subplots of size 1.4 m x 0.7 m, one of which received light addition and one was unlighted. Plant community data were collected from each subplot from a permanent quadrat (see below "sampling strategy"). Therefore, subplots were nested within plots that were nested within blocks. There were 80 subplots/quadrats in the study and five replicates per treatment combination (considering all four treatments). As climate was never significant for Shannon diversity and richness (i.e. the response variables of our main interest), we pooled the data across the climate treatment (ten replicates per treatment combination).
Research sample	A permanent quadrat of size 0.5 x 0.5 m which was studied for aerial percentage cover for all plant species occurring in the quadrat. This size is a standard size in vegetation analyses and was the largest size that fit to our experimental subplots. Using data on all individual species occurring in the quadrats, we calculated 1) species richness (i.e., number of species), 2) Shannon diversity, 3) total cover (i.e. the summed cover of all plant species), and 4) litter cover, for each individual quadrat, and used these variables as response variables in our statistical models. Quadrats received different manipulations in a full-factorial way (see Study description).
Sampling strategy	The number of grazed pastures was determined by Global Change Experimental Facility (Schädler, M. et al. 2019 Ecosphere) within which our experiment was conducted. Our sample size, plots, subplots and quadrats (sample units) are of typical size when manipulating and examining plant communities in ecological research.
Data collection	Anu Eskelinen investigated the permanent quadrats and estimated visually percentage cover for all plant species occurring in the quadrats.
Timing and spatial scale	The experiment was established in May 2017. In 2017, we sampled the quadrats in peak biomass (mid-July) i.e., when plants were fully grown. In this year, we sampled the quadrats later than the in 2019 because vegetation in all plots and surrounding areas was trimmed to 5 cm height at time of the establishment of the experiment at the end of May, and it took time for vegetation to reach its maximum biomass. In 2019, we sampled the plots at the end of May - the beginning of June, also when vegetation was in its peak biomass.
Data exclusions	No data were excluded.
Reproducibility	We have used standard experimental and sampling methods in community ecology and have carefully reported how our unique 'eDiValo' field experiment and the experimental manipulations were executed and how the sampling was done to make sure that our experiment can be reproduced. Scripts used to analyze data and make figures are available via Dryad Data repository. For further information about the experiment, please see Figure 1 and Methods.
Randomization	The experimental plots were randomly allotted to the following treatments: fertilization, no fertilization, herbivore exclusion, herbivore exclusion and fertilization. The subplots within a plot were randomly allotted to light addition or no light addition treatments.
Blinding	Blinding was not relevant to our field study. Treatments in the field are clearly visible (e.g. lamps, herbivore exclosures).

Did the study involve field work? ☒ Yes ☐ No

Field work, collection and transport

Field conditions	Long-term mean annual precipitation in the area is 489 mm and mean annual temperature is 8.9 degrees C. During 2018-2019 Europe experienced a record-setting drought that was especially severe in 2018. The mean annual precipitation at our study site in Bad Lauchstädt research station was 254 mm in 2018 and 353 mm in 2019. 2017 was a more normal year with mean annual precipitation of 403 mm. Mean annual temperatures were also above average: 2017, 10.5 degrees C; 2018, 10.8 degrees C; 2019, 11.2 degrees of C.
Location	Our study site is located at Bad Lauchstädt Field Research Station, in Bad Lauchstädt, Germany (51°22060 N, 11°50060 E), at 118 m a.s.l. The Field Research Station belongs to Helmholtz Centre for Environmental Research-UFZ.
Access & import/export	Our experiment was conducted in Global Change Experimental Facility (GCEF), meant for experimental research. No permits were needed. Sampling followed standard practices and followed all local and national laws.

Disturbance	Our experiment was conducted in a field experimental facility (GCEF; see above), which is dedicated to experimental research. We disturbed the vegetation in the area as little as possible by using the same paths when walking to the experiment. Roads established during the construction of GCEF lead very close (~20 m) to the blocks within which our eDiValo experiment was located, and not much walking was involved. Disturbance at the experiment was therefore minimal.

Reporting for specific materials, systems and methods

We require information from authors about some types of materials, experimental systems and methods used in many studies. Here, indicate whether each material, system or method listed is relevant to your study. If you are not sure if a list item applies to your research, read the appropriate section before selecting a response.

Materials & experimental systems

n/a	Involved in the study
☒	☐ Antibodies
☒	☐ Eukaryotic cell lines
☒	☐ Palaeontology and archaeology
☒	☐ Animals and other organisms
☒	☐ Human research participants
☒	☐ Clinical data
☒	☐ Dual use research of concern

Methods

n/a	Involved in the study
☒	☐ ChIP-seq
☒	☐ Flow cytometry
☒	☐ MRI-based neuroimaging
